# Three-dimensional analysis of the tibial resection plane relative to the arthritic tibial plateau in total knee arthroplasty

**DOI:** 10.1186/s40634-017-0099-z

**Published:** 2017-08-08

**Authors:** J. Michael Johnson, Mohamed R. Mahfouz, Mehmet Rüştü Midillioğlu, Alexander J. Nedopil, Stephen M. Howell

**Affiliations:** 1 TechMah LLC, Knoxville, TN 37922 USA; 2 Department of Mechanical, Aerospace and Biomedical Engineering, The University of Tennessee, 307 Perkins Hall, 1506 Middle Drive, Knoxville, TN 37996 USA; 3 Department of Biomedical Engineering, University of California, Davis, CA 95616 USA; 4 Department of Orthopaedics, University of California, Davis, CA 95817 USA; 5 Department of Orthopaedics, Gazi Mustafa Kemal State Hospital, Ankara, 06710 Turkey

**Keywords:** Total knee arthroplasty, Arthritic tibia, Kinematics, Tibial resection, Three-dimensional surgical planning, Tibial plateau, Tibial joint line, Kinematic Alignment

## Abstract

**Background:**

Kinematically aligned total knee arthroplasty strives to correct the arthritic deformity by restoring the native tibial joint line. However, the precision of such surgical correction needs to be quantified in order to reduce recuts of the resection and to design assisting instrumentation. This study describes a method for novel three-dimensional analysis of tibial resection parameters in total knee arthroplasty. Pre-operative versus post-operative differences in the slopes of the varus-valgus and flexion-extension planes and the proximal-distal level between the tibia resection and the arthritic tibial joint line can reliably be measured using the three-dimensional models of the tibia and fibula. This work uses the proposed comparison method to determine the parameters for resecting the tibia in kinematically aligned total knee arthroplasty.

**Methods:**

Three-dimensional shape registration was performed between arthritic surface models segmented from pre-operative magnetic resonance imaging scans and resected surface models segmented from post-operative computed tomography scans. Mean, standard deviation and 95% confidence intervals were determined for all measurements.

**Results:**

Results indicate that kinematically aligned total knee arthroplasty consistently corrects the varus deformity and restores the slope of the flexion-extension plane and the proximal-distal level of the arthritic tibial joint line. The slope of the varus-valgus plane is most precisely associated with the overall arthritic slope after approximately 3° of correction and the posterior slope is biased towards the overall arthritic plateau, though less precisely than the varus correlation.

**Conclusions:**

Use of this analysis on a larger population can quantify the effectiveness of the tibial resection for correcting pathologies, potentially reduce imprecisions in the surgical technique, and enable development of instrumentation that reduces the risk of resection recuts. The kinematic alignment technique consistently corrects varus deformities.

## Background

Varus alignment of the tibial component is associated with tibial loosening in total knee arthroplasty (TKA) (Jeffery et al. 1991; Windsor et al. 1989). Kinematically aligned (KA) TKA strives to correct the arthritic deformity and restore the native varus-valgus (V-V) and flexion-extension (F-E) planes and the proximal-distal (P-D) level of the tibial joint line. Despite work assessing the promising post-operative outcomes of KA TKA (Dossett et al. 2012; Howell et al. 2013a), a straightforward femoral resection technique (Howell et al. 2017), and postoperative limb alignment (Dossett et al. 2014; Ji et al. 2016), there remain uncertainties regarding the precision for placing the tibial component. This aim of this study is to better understand the three-dimensional orientation of the tibial resection to allow surgeons to achieve reproducible outcomes. Providing quantification of the resection may also provide an avenue to reduce recuts of the resection and design assisting instrumentation.

## Methods

### Aim, Design and Setting

This study introduces and reports the repeatability of a novel three-dimensional (3D) analysis for computing pre- versus post-operative differences in the slopes of the V-V and F-E planes and the P-D level between the tibial component and the arthritic tibial joint line. The aim of this work is to utilize the 3D analysis method to quantify the surgical placement parameters for the tibial component during KA TKA in pathologically varus knees. A first step in this analysis is the use of previously described techniques to construct 3D images of the arthritic knee from magnetic resonance imaging (MRI) scans (Eckstein et al. 2008; Stirling et al. 2016; Wirth et al. 2011), a process with applications which range from analyzing osteoarthritic progression to patient-specific surgical instrumentation. After implantation of a TKA, repeat joint imaging with MRI is no longer feasible due to distortion from the metallic components. Instead, 3D images of the reconstructed knee can be generated from computed tomography (CT) scans (Kim et al. 2009; Lützner et al. 2008), which are used to measure component positions and resection planes (Hirschmann et al. 2011; Plaskos et al. 2002). The 3D analysis presented was utilized to retrospectively determine pre- versus post-operative differences in the slope of the V-V and F-E planes and the P-D level between the tibial component and the arthritic tibia with a varus deformity in an osteoarthritic patient population after treatment with KA TKA.

#### Surgical Technique

All subjects in this study were treated with KA TKA. The following sequence of surgical steps, caliper measurements and adjustments were used to achieve quality assurance in kinematically aligning the femoral and tibial components coincident to the native joint lines (Howell et al. 2017; Howell et al. 2013b). Step 1: Identify the distal femoral condyles with cartilage wear. Step 2: Remove partial cartilage wear to bone. Step 3: Apply a distal femoral referencing guide that compensates 2 mm thickness when cartilage is worn on the distal medial femoral condyle in the varus knee, and 2 mm thickness when cartilage is worn on the distal lateral femoral condyle in the valgus knee. Step 4: Measure the thicknesses of the distal femoral resections with a caliper. Step 5: Adjust the thickness of each resection to match the thickness of the condyles of the femoral component after compensating for cartilage wear and kerf to within ± 0.5 mm. When the distal resection is 1–2 mm too thin, angle the blade in the saw slot and recut the bone using the ~1 mm thickness of the sawblade as a gauge. When the distal resection is 1–2 mm too thick, apply a 1 or 2 mm thick washer on the peg of the 4-in-1 chamfer block, which shims a corrective gap between the condyle of the femoral component and the distal femur. Step 6: Position the 4-in-1 chamfer block by drilling holes through a posterior femoral referencing guide set at 0° rotation. Step 7: Measure the thicknesses of the posterior femoral resections with a caliper before making the anterior and chamfer cuts. Step 8: Adjust the thickness of each resection to match the thickness of the condyles of the femoral component after compensating for cartilage wear and kerf to within ± 0.5 mm. When a posterior femoral resection is 1–2 mm too thick or too thin, eccentrically elongate the pin hole in the direction of the correction and translate the 4-in-1 chamfer block as needed. Step 9: Secure the chamfer block in the corrected position with compression screws. Step 10: Make the anterior resections and chamfer femoral resections. These caliper measurements and adjustments are quality assurance steps that align the femoral component coincident to the native distal and posterior femoral joint lines. Step 11: Remove medial and lateral osteophytes. Step 12: Apply a conventional extramedullary tibial resection guide to the ankle and place an angel wing in the saw slot of the guide. Step 13: Adjust the varus-valgus angle of the tibial resection guide until the saw slot parallels the proximal tibial articular surface and the angel wing parallels the slope of the medial tibia after compensating for wear. Step 14: Resect the proximal tibia. Step 15: Measure the thickness of the medial and lateral tibial condyles at the base of the tibial spines. When one tibial condyle is thinner than the other by 1 mm or more, expect tightness in that compartment and slackness in the other when assessing varus-valgus laxity with trial components with the knee in full extension. Step 16: When asymmetric laxity is observed, use a 2° varus or valgus recut guide to fine-tune the tibial resection until the laxity is 1° or less in full extension like the native knee (Roth et al. 2015). These caliper measurements and adjustments are quality assurance steps that the tibial component is coincident to the native proximal tibial joint line and co-aligns the components to the three rotational axes of the native knee (Howell et al. 2017; Howell et al. 2013b).

### Dataset

**Fig. 1:**
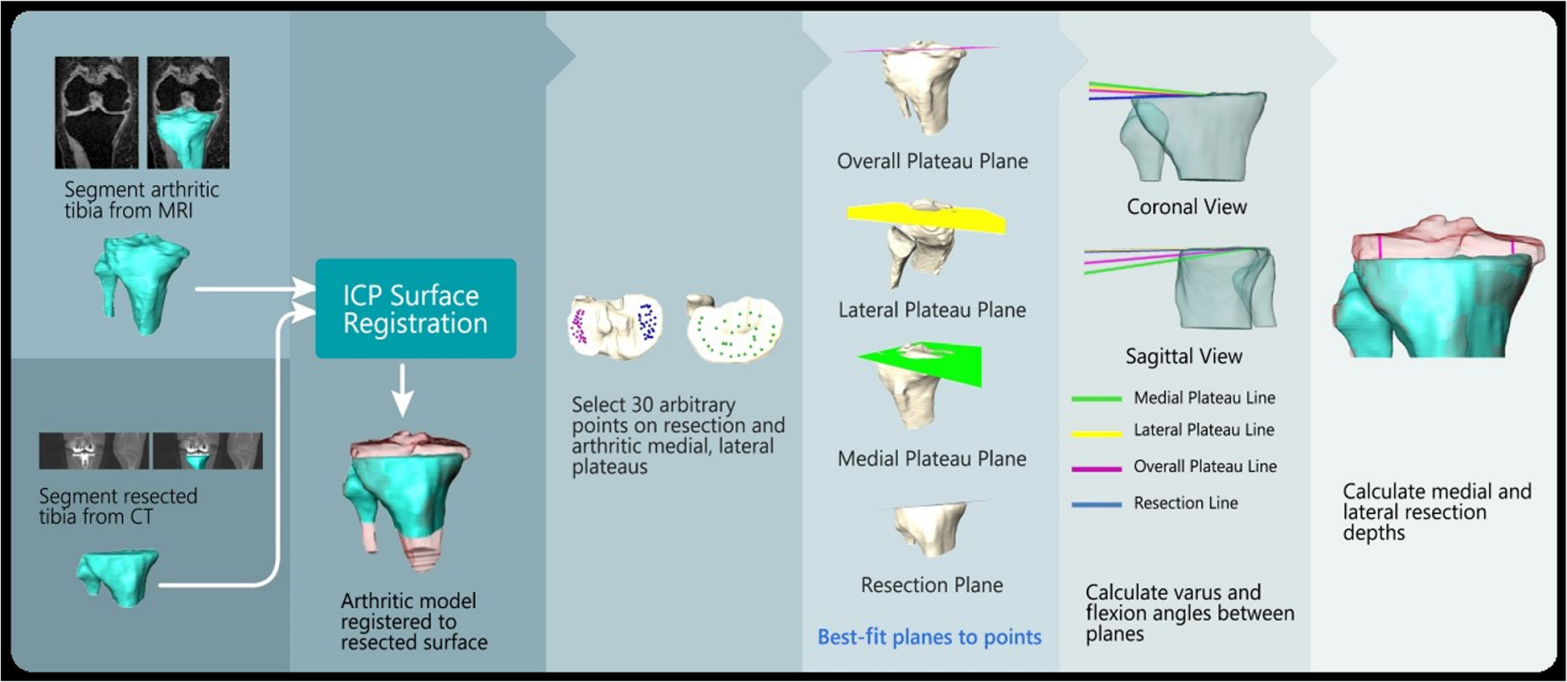
Registration process of surface models, point selection, plane fitting, and calculations. [The registration process for the various steps are represented. Panels one and two represent pre-operative MRI and post-operative resected CT surface models; panels three and four represent point selection and plane fitting, respectively; panels five and six represent calculations.]

Figure 1 presents a flowchart for the overall method for measuring the tibial resection parameters utilizing three-dimensional shape registration, point selection, plane fitting, and quantifying KA TKA restoration. Fifteen patients with a varus osteoarthritic knee were randomly selected for this study (IRB approval # 895814–1). Each patient had a pre-operative MRI scan of the osteoarthritic knee and was subsequently treated with a kinematically aligned TKA by a single surgeon. A post-operative CT scan was performed. From the pre-operative MRI of the arthritic knee, the tibia bone, tibia cartilage, and fibula were segmented using commercial software (Avizo version 8.1.0, FEI, 5350 NE Dawson Creek Drive, Hillsboro, Oregon 97124 USA). From the post-operative CT of the reconstructed knee, the resected tibia, fibula, and implant component were segmented. The quality of all segmentations were verified by an expert having over 5 years of experience segmenting the anatomy. From each segmentation, 3D surface models were created using a marching cubes algorithm (Hege et al. 1997). Additionally, the size of the tibial baseplate and the thickness of the insert were recorded for each case (Persona CR, Zimmer-Biomet, Inc.,345 East Main St, Warsaw, IN 46580 USA).

MRI scanning parameters were sagittal fast spoiled gradient echo with fat saturation (FSPGR fat sat) having 2 mm slice thickness reconstructed to 1 mm. Scans were of the entire joint, centered at the joint line, including tibial tubercle and distal femoral shaft. A 1.25-mm thick axial computed tomography (CT) scan of the hip, knee, and ankle was performed on each patient before discharge using a previously described protocol (Nedopil et al. 2016; Nedopil et al. 2013).

#### Registration

For each patient, the transformation used to register the arthritic MRI surfaces to the resected CT surfaces was calculated by registering the combined models from the MRI of the tibia and fibula surfaces with the CT tibia and fibula surfaces. The rigid registration process involved an initial alignment of the surface centroids followed by an iterative closest point (ICP) step (Besl & McKay 1992) to refine results. The ICP step used stopping criteria of relative root-mean-squared difference between successive iterations below 0.001 mm and a maximum number of 150 iterations for maximum resolution.

Given differences between MRI and CT surfaces due to the resection and different size fields of view, the registrations were performed only on those portions of each surface that was within a common region of interest (ROI). The ROI was selected to be immediately proximal to the distal ends of both the MRI and CT models and extended proximally to the resection plane, though the resection plane was not included. Defining such an ROI for registration is justified to avoid misalignment due to different length surfaces and to avoid bias due to the resection surface. The registration root mean square error (mm) and standard deviation (mm) within the ROI between the pre-operative and resected tibia was calculated for each patient as a point-to-point error in distance between the two surface models. The closest point root mean square error (RMSE) and standard deviation for surface registration was 0.69 mm ± 0.42 mm. Maximum RMSE was 0.85 mm. This data is reported in Table 1.

**Table 1:** Registration error and standard deviation between resected tibia and pre-operative tibia resected tibia and pre-operative tibia

Subject #	RMS Error (mm)	Standard Dev (mm)
1	0.59	0.38
2	0.75	0.50
3	0.58	0.36
4	0.50	0.31
5	0.66	0.39
6	0.81	0.55
7	0.84	0.54
8	0.63	0.36
9	0.65	0.36
10	0.65	0.36
11	0.85	0.55
12	0.69	0.39
13	0.68	0.44
14	0.68	0.40
15	0.75	0.46

[Table 1 measurements within the registration region of interest represent 15 patients.]

#### Landmarks

A primary objective of this work is to determine the difference in orientation of the post-operative tibial resection plane relative to the orientation of the planes of the pre-operative arthritic proximal tibial joint line in the osteoarthritic knee with a varus deformity. To do this, a coordinate system was defined on the tibia to compute the differences in the orientation of the V-V and F-E planes and the P-D level between the tibial resection and the proximal joint line of the arthritic tibia.

From the arthritic tibia, the superior-inferior (SI) direction was defined by a line connecting the center between the tibial spines to the center of the distal end of the tibia. The anterior-posterior (AP) direction was defined by a line connecting the center of the insertion of the posterior collateral ligament to the medial third of the tibial tubercle. The medial-lateral (ML) direction was defined as the cross-product of the AP and SI directions. Resection measurements were referenced from three best-fit planes to the articular surfaces of the arthritic joint line of the lateral compartment (lateral plateau plane), medial compartment (medial plateau plane), and overall joint surface (overall plateau plane) of the arthritic tibia, as shown in Fig. 1.

The planes of the medial and lateral compartments were best-fit to 30 points selected by a user on the articular surfaces of the medial and lateral tibial condyles. The plane of the overall joint surface was determined to be the best-fit plane to all 60 selected points. All landmarks, planes, and directions defined in the MRI coordinate system were transformed to the CT coordinate system using transformations calculated during the MRI and CT registration processes. From the CT model, the plane of the tibial resection was best-fit to 30 points on the tibia just distal from the tibial component.

**Fig. 2:**
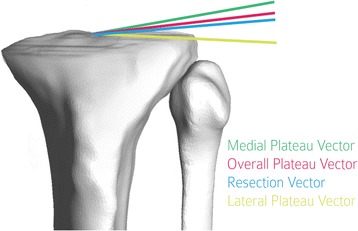
Relative varus orientation measured after projection on coronal plane (Subject 11). [Directions are shown eminating from a single point to facilitate visualization of measurements.]

The differences between the varus orientation of the resection plane relative to the pre-operative tibial planes were calculated by projecting the ML direction onto the resection plane and each pre-operative tibial plane. Each projected ML vector was subsequently projected onto the coronal plane. The coronal plane was defined as the plane containing the mid-point of the tibial spines, with normal direction equal to the cross-product of the ML and SI directions. The angle between these coronal projections defines the difference in varus orientation between the resection plane and the pre-operative tibial plane. Figure 2 depicts the coronal orientation of the medial, lateral and overall plateau vectors and the resection vector.

**Fig. 3:**
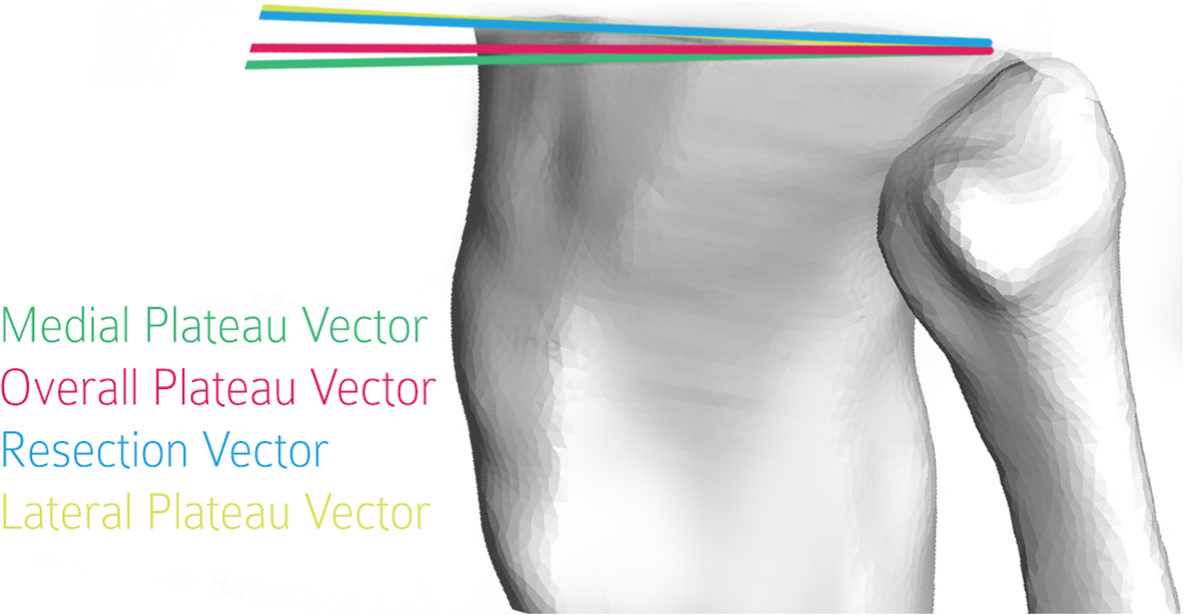
Relative flexion measured after projection on sagittal plane (Subject 7). [As in Fig. 2, the directions are shown eminating from a single point to facilitate visualization of measurements.]

The relative flexion for each pre-operative plane was calculated by projecting the AP direction onto the resection plane and the pre-operative tibial plane. Each projected AP vector was projected onto the sagittal plane (defined as the plane passing through the mid-point of the tibial spine with normal direction equal to the ML direction). The angle between these sagittal projections defines the difference in flexion between the resection and the respective tibial plateau. Figure 3 depicts the sagittal orientation of the medial, lateral and overall plateau vectors and the resection vector.

**Fig. 4:**
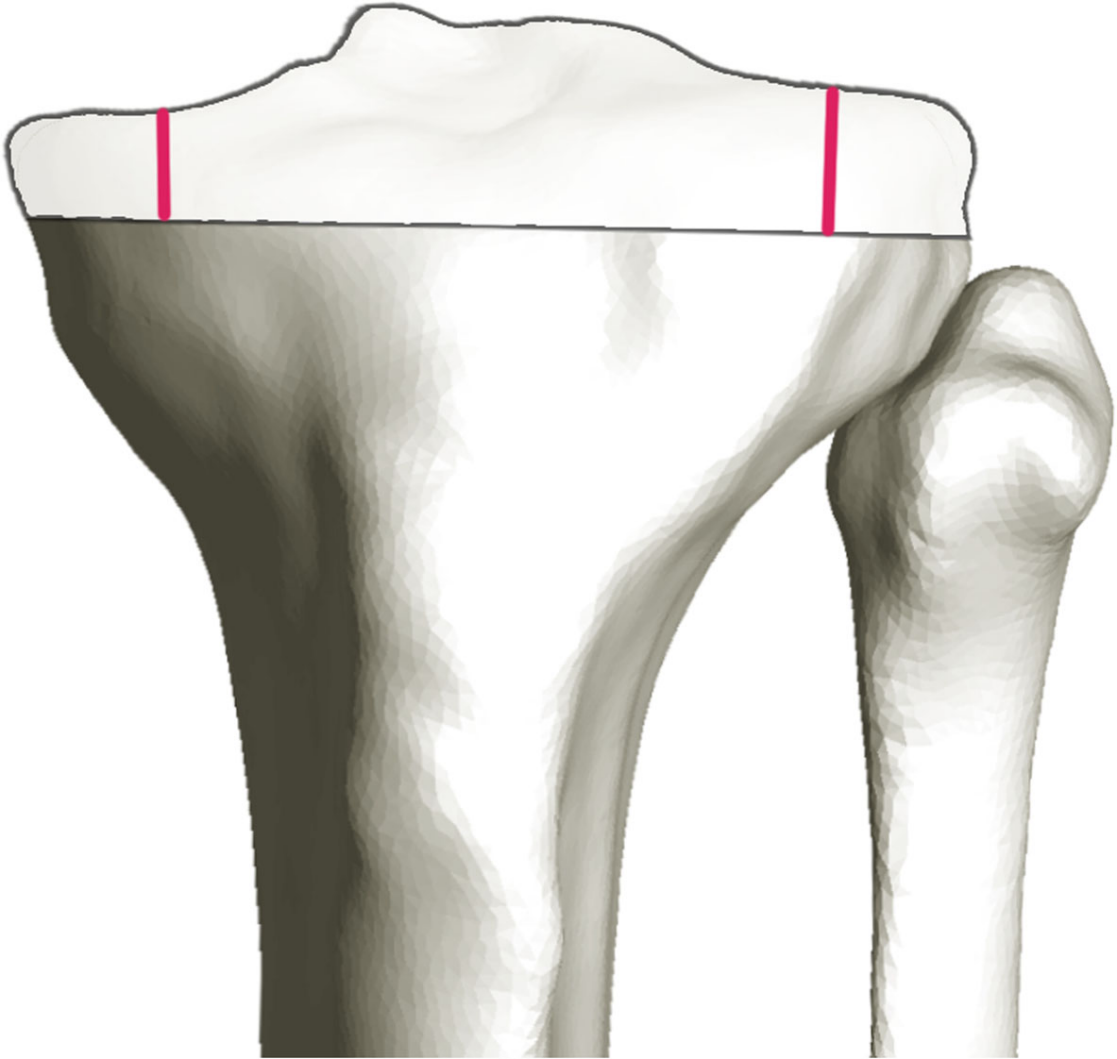
Resection depth (Subject 3). [Transparent anatomy is the MRI surface model; opaque anatomy is the resected CT surface model.]

Finally, the resection depths for the medial and lateral tibial condyle were calculated as the mean distance between the selected points on each condyle and the resection plane. An example is shown in Fig. 4.

### Statistical Methods

Mean, standard deviation, and 95% confidence intervals were determined for all measurements. A repeatability study for the calculation of the planes for a single case among five operators is reported in Table 2. Results of intraoperator variability, across 10 repeat measurements of the same case used in the interoperator study are shown in Table 3. Measurements describe the angular differences in V-V (+ varus) and F-E (− flexion) planes and the translational difference in P-D (+ distal) level of the overall, medial, and lateral compartments of the articular surface of the arthritic tibia to the resection plane. Negative flexion indicates a pre-operative plane which is less flexed than the resection plane. Positive varus indicates a pre-operative plane which is more varus than the resection plane.

**Table 2:** Plane repeatability study for point selection process among five operators for a single patient case

	Flexion vs. Resection (degrees)			Varus vs. Resection (degrees)			Depth (mm)		
Operator #	Medial	Lateral	Overall	Medial	Lateral	Overall	Medial	Lateral	Overall
1	−8.9	−1.9	−5.2	7.5	6.3	4.5	6.1	9.3	7.7
2	−7.0	−2.4	−4.5	8.0	2.3	4.7	5.8	9.6	7.7
3	−7.3	0.1	−3.9	7.7	5.6	4.2	5.9	9.2	7.6
4	−7.9	−1.8	−5.1	7.9	−0.2	4.8	5.8	9.5	7.6
5	−6.6	−1.8	−4.6	9.4	3.3	3.5	6.6	9.0	7.8
Mean	−7.5	−1.6	−4.7	8.1	3.5	4.3	6.0	9.3	7.7
Standard Dev	0.9	1.0	0.5	0.8	2.6	0.5	0.3	0.2	0.1

**Table 3:** Plane repeatability study for point selection process for single operator

	Flexion vs. Resection (degrees)			Varus vs. Resection (degrees)			Depth (mm)		
Operator #	Medial	Lateral	Overall	Medial	Lateral	Overall	Medial	Lateral	Overall
1	−8.6	−1.8	−5.0	9.5	4.8	4.3	6.3	9.3	7.8
2	−8.0	−2.7	−6.1	9.3	−2.8	4.8	6.4	9.5	8.0
3	−7.5	−2.8	−5.3	9.7	3.8	4.5	6.6	9.3	7.9
4	−8.3	−1.2	−5.7	8.8	1.2	4.4	6.4	9.4	7.9
5	−8.0	−2.2	−5.8	8.2	5.8	4.7	6.2	9.4	7.8
6	−7.1	−3.5	−5.9	9.7	−0.5	4.6	6.6	9.4	8.0
7	−8.3	−2.7	−6.0	9.7	0.6	4.6	6.5	9.4	7.9
8	−7.8	−2.5	−6.3	8.5	0.2	4.8	6.4	9.5	7.9
9	−7.8	0.1	−5.5	9.2	1.4	4.3	6.6	9.5	8.0
10	−7.6	−2.1	−6.0	8.9	0.2	4.7	6.2	9.5	7.9
Mean	−7.9	−2.1	−5.8	9.1	1.5	4.6	6.4	9.4	7.9
Standard Dev	0.5	1.0	0.4	0.5	2.6	0.2	0.1	0.1	0.1

## Results

Tables 4 and 5 summarize the differences in the relative posterior slope and the relative varus slope in degrees between each of the calculated planes. The mean and standard deviation are reported for fifteen patients, where the abbrevations are: medial condyle (“M”), lateral condyle (“L”), overall plateau (“O”), and resection plane (“R”). A positive value indicates that the first variable has more slope than the second. (For example, an O-R value of −1.1° for the difference in F-E orientation in Table 4 indicates the overall plateau has 1.1° *more anterior slope* than the resection plateau. An M-L value of 12.7° for the difference in V-V slope in Table 5 indicates the medial plateau is 12.7° more varus than the lateral plateau.)

**Table 4:** Difference in F-E orientation between each pair of calculated planes for 15 patients

	F-E orientation difference (degrees)					
Subject #	O-R	M-R	L-R	M-L	M-O	L-O
1	−1.1	−1.4	−0.8	−0.6	−0.3	0.3
2	0.5	−2.7	7.1	−9.8	−3.2	6.6
3	−2.6	−2.2	−3.8	1.6	0.4	−1.2
4	2.2	1.5	2.7	−1.3	−0.7	0.6
5	−1.3	−5.1	2.8	−7.9	−3.7	4.2
6	1.2	0.7	−0.8	1.5	−0.5	−2.0
7	−2.6	−4.3	0.3	−4.6	−1.7	2.9
8	0.4	1.3	−1.1	2.5	0.9	−1.5
9	4.3	4.1	3.7	0.3	−0.2	−0.6
10	−2.0	−1.3	−2.1	0.8	0.7	−0.1
11	0.0	−3.4	2.6	−6.0	−3.4	2.6
12	−3.9	−7.3	0.1	−7.3	−3.3	4.0
13	5.0	2.7	5.7	−3.0	−2.2	0.8
14	−2.1	−5.2	0.7	−6.0	−3.1	2.8
15	−1.1	−2.7	0.8	−3.5	−1.6	1.9
Mean	−0.2	−1.7	1.2	−2.9	−1.5	1.4
Standard Dev	2.5	3.2	2.9	3.9	1.6	2.4

**Table 5:** Difference in V-V orientation between each pair of calculated planes for 15 patients

	V-V orientation difference (degrees)					
Subject #	O-R	M-R	L-R	M-L	M-O	L-O
1	3.7	2.9	5.7	−2.8	−0.9	1.9
2	2.4	9.6	−3.1	12.7	7.3	−5.4
3	3.4	6.3	−2.8	9.1	2.9	−6.3
4	3.5	11.4	3.6	7.8	7.9	0.0
5	4.9	5.5	−3.8	9.3	0.7	−8.7
6	1.9	5.4	−9.3	14.6	3.4	−11.2
7	3.1	8.0	−5.8	13.7	4.9	−8.9
8	1.8	3.6	2.6	1.0	1.8	0.8
9	1.5	2.1	−0.6	2.7	0.6	−2.1
10	1.7	3.9	2.6	1.3	2.2	0.9
11	2.3	5.7	−6.4	12.1	3.4	−8.8
12	4.2	7.7	5.6	2.1	3.5	1.4
13	−0.5	4.4	−9.6	14.0	4.9	−9.1
14	2.4	9.7	−8.0	17.7	7.3	−10.4
15	2.1	2.6	4.4	−1.8	0.5	2.3
Mean	2.6	5.9	−1.7	7.6	3.4	−4.2
Standard Dev	1.3	2.8	5.5	6.6	2.7	5.1

Thus, the orientation of the V-V plane of the tibial resection averaged 2.6° ± 1.3° more valgus than the overall plane of the arthritic tibia (95% CI 3.3° to 1.8°), 5.9° ± 2.8^o^ more valgus than the plane of the medial or worn compartment (4.4° to 7.5°), and 1.7° ± 5.5^o^ more varus than the plane of the lateral or unworn compartment (4.7° more varus to 1.4^o^ more valgus). The orientation of the F-E plane of the tibial resection averaged 0.2° ± 2.5° less flexed (i.e., less posterior slope) than the overall plane of the arthritic tibia (95% CI 1.6° less flexed to 1.2° more flexed), 1.7° ± 3.2° less flexed than the plane of the medial or worn compartment (3.5° less flexed to 0.1° more flexed), and 1.2° ± 2.9° more flexed than the plane of the lateral or unworn compartment (0.4° less flexed to 2.8° more flexed). The resection depth of the tibia averaged 6.8 mm ± 0.9 mm distal from the plane of the medial or worn compartment (6.3 mm to 7.3 mm) and 9.1 mm ± 1.1 mm distal from the plane of the lateral or unworn compartment (8.4 mm to 9.7 mm). When the thickness of the tibial baseplate and insert were added to the tibial resection, the level of the dwell points of the insert averaged 3.6 mm ± 1.1 mm more proximal from the arthritic medial side (3.1 mm to 4.2 mm) and 1.4 mm ± 1.1 mm more proximal from the non-arthritic lateral side (0.8 mm to 2.0 mm).

Table 6 reports the resection depth and the difference in the P-D level after accounting for the component in the medial, lateral and overall planes for fifteen patients. The difference in the P-D level is calculated as the resection depth subtracted from the construct (baseplate + liner) thickness. A positive value for difference in the P-D level indicates that the construct is thicker than the measured resection depth. Illustrative examples of various subjects are shown in Figs. 2, 3, and 4 to facilitate visualization of measurements.

**Table 6:** Resection depth and difference in the P-D level for 15 patients (in millimeters)

	Resection Depth (mm)			Difference in the P-D level (mm)		
Subject #	Medial	Lateral	Overall	Medial	Lateral	Overall
1	7.1	10.6	8.9	3.9	0.4	2.1
2	6.7	8.7	7.7	3.3	1.3	2.3
3	8.2	10.9	9.6	1.8	−0.9	0.4
4	6.1	9.3	7.7	3.9	0.7	2.3
5	6.2	10.6	8.4	5.8	1.4	3.6
6	6.6	8.3	7.5	3.4	1.7	2.5
7	7.1	9.9	8.5	4.9	2.1	3.5
8	6.8	8.4	7.6	3.2	1.6	2.4
9	7.6	9.0	8.3	2.4	1.0	1.7
10	7.3	8.5	7.9	2.7	1.5	2.1
11	5.3	7.2	6.2	4.7	2.8	3.8
12	5.9	9.2	7.6	4.1	0.8	2.4
13	8.5	8.5	8.5	3.5	3.5	3.5
14	5.4	7.3	6.4	4.6	2.7	3.6
15	7.5	9.4	8.4	2.5	0.6	1.6
Mean	6.8	9.1	7.9	3.6	1.4	2.5
Standard Dev	0.9	1.1	0.9	1.1	1.1	0.9

[Table 6 measurements were taken after accounting for the medial condyle, lateral condyle and overall plateau components.]

## Discussion

This study proposes a reliable 3D method for analyzing tibial resection planes post-operatively relative to the pre-operative arthritic tibial joint line. The authors used this method to quantify the placement of the tibial component in varus knees treated with KA TKA. While this work focuses solely on placement of the tibia, an objective of KA TKA is appropriate post-operative joint alignment. Although the femur was not considered in this work, the joint alignment outcomes of KA TKA have been reported previously (Dossett et al. 2014; Ji et al. 2016). This initial work on the tibia suggests treatment with KA TKA produces a consistent correction of the varus deformity and consistent restoration of the F-E slope and the P-D level of the arthritic joint line. It is understood that the tibiofemoral forces and ligament balance are sensitive to the flexion and extension gap in TKA (Jeffcote et al. 2007; Gu et al. 2014). A secondary result of this limited investigation is some visibility into the KA TKA anatomical references for establishing the resection parameters. Specifically, the V-V orientation is most precisely associated with the overall arthritic slope corrected approximately 3° and the F-E plane is biased towards the overall arthritic plateau, though the F-E plane is not as precisely correlated to pre-operative features. The relative lack of precision in setting the F-E plane may be due to the more difficult sight-lines in the operating room when assessing the F-E plane, which requires estimation of the slope in the sagittal plane and may not be readily visible in many cases. These types of resection analyses may help to refine surgical technique, especially when correlated with clinical follow-up metrics.

As the body of research improves, quantifying component placement parameters provides important avenues for more appropriate virtual surgical planning and technology-assisted arthroplasty, especially in patients treated with KA TKA. Comparing the results in this work to previously reported cartilage loss patterns in varus osteoarthritic knees (Johnson & Mahfouz 2016; Eckstein et al. 2010), it is reasonable to presume that the correction of varus deformity in the tibia will result in a larger proximal shift in the P-D level of the medial compartment than a lateral shift (3.6 mm ± 1.1 mm medial vs 1.4 mm ± 1.1 lateral) to account for cartilage and bone loss that is predominantly present in the medial compartment. Eckstein has reported that the central medial tibia compartment and external medial tibial compartment are most susceptible to cartilage loss as osteoarthritis progresses-indicating the predominant change in orientation of the tibial plateau will be an increased varus slope and that the F-E orientation may be less affected by pathological changes (Eckstein et al. 2010). In this work, we see that KA TKA corrected varus deformity (measured as a positive difference in V-V orientation between the medial plateau and the resection plane) in 15 of 15 clinical cases, but the difference in the overall F-E orientation showed no such consistency. This is consistent with current understanding of osteoarthritic deformities in varus knees.

Limitations of this study include imprecision in measurements due to point selection subjectivity, lack of meniscus in determination of the orientation of the articular surface, and latent registration error.

## Conclusions

While outcomes for patients receiving KA TKA are promising, determining the parameters for correcting tibial pathological deformation remains subjective. Therefore, it is important to develop precise methodology and optimal implementation of these novel procedures in the operating room. While the methods presented here are impractical for routine intraoperative assessment, the primary aim of this study was to utilize the 3D retrospective analysis to quantify the tibial resection parameters used in varus knees treated with KA TKA. The methods described provided reproducible numerical results that matched well with current understanding of varus osteoarthritic disease deformities. While the sample size was limited in this work, the process proposed here, when used on a larger population, can help quantify the precision of tibial resection for correcting varus and valgus pathologies, potentially reduce imprecisions in the surgical technique, and enable development of instrumentation that reduces the need for resection recuts. Understanding appropriate resection parameters also provides avenues for integration of assisting technologies into the KA TKA workflow, such as robotic navigation or patient-specific instrumentation, which are predicated on an understanding of the numerical targets prior to guiding resections.

Future work should explore relationships between the meniscus and the tibial joint line and include subjects with different malalignment. Where possible, resection measurements should be correlated to clinical follow-up metrics.

## Abbreviations

“L”: Lateral Condyle
“M”: Medial Condyle
“O”: Overall Plateau
“R”: Resection Plane
3D: Three-Dimensional
AP: Anterior-Posterior
CT: Computed Tomography
F-E: Flexion-Extension
FSPGR fat sat: Fast Spoiled Gradient Echo with fat saturation
ICP: Iterative Closest Point
KA: Kinematically Aligned
ML: Medial-Lateral
MRI: Magnetic Resonance Imaging
P-D: Proximal-Distal
RMSE: Root Mean Square Error
ROI: Region of Interest
SI: Superior-Inferior
TKA: Total Knee Arthroplasty
V-V: Varus-Valgus
